# Complete Genome Sequence of Classical Swine Fever Virus Strain JSZL, Belonging to a New Subgenotype, 2.1d, Isolated in China in 2014

**DOI:** 10.1128/genomeA.00833-15

**Published:** 2015-08-20

**Authors:** Hongliang Zhang, Liping Feng, Chunxiao Liu, Jiazeng Chen, Chaoliang Leng, Yun Bai, Jinmei Peng, Tongqing An, Xuehui Cai, Xufu Yang, Zhijun Tian, Guangzhi Tong

**Affiliations:** a State Key Laboratory of Veterinary Biotechnology, Harbin Veterinary Research Institute, Chinese Academy of Agricultural Sciences, Harbin, China; b North Guangdong Collaborative Innovation and Development Center of Pig Farming and Disease Control, Shaoguan University, Shaoguan, China; c Shanghai Veterinary Research Institute, Chinese Academy of Agricultural Sciences, Shanghai, China

## Abstract

The complete genome sequence of classic swine fever virus (CSFV) strain JSZL was determined in this study. JSZL was originally isolated from an immune pig farm in Jiangsu Province, China. JSZL is more closely related to subgenotype 2.1b than to 2.1a and 2.1c. Importantly, JSZL was classified into a new subgenotype, 2.1d.

## GENOME ANNOUNCEMENT

Classical swine fever (CSF), listed with the Office International des Epizooties (OIE), is one of the most highly contagious and fatal diseases of swine. Classical swine fever virus (CSFV), the causative agent of CSF, is a member of the genus *Pestivirus* of the family *Flaviviridae*, which also contains bovine viral diarrhea virus types I and II (BVDV I and II) and border disease virus (BDV) (1, 2). CSFV is a single positive-stranded RNA virus, whose genome is approximately 12.3 kb in length, and it comprises a single long open reading frame (ORF) that encodes a polyprotein composed of 3,898 amino acids, flanked by two noncoding regions at the 5′ UTR and 3′ UTR. Based on the E2 gene and a partial nonstructural 5B (NS5B) gene, CSFV isolates collected in 14 countries from 1945 to 1994 were classified into two genotypes and five subgenotypes (3). Next, CSFV isolates were further classified into three genotypes (1–3) and 10 subgenotypes (1.1, 1.2, 1.3, 2.1, 2.2, 2.3, 3.1, 3.2, 3.3, and 3.4) (4). Isolates of subgenotype 2.1 were further classified into 2.1a and 2.1b (5, 6). In recent years, isolates of subgenotype 2.1c and 1.4 emerged (7, 8).

The novel CSFV strain JSZL belongs to a subgenotype, 2.1d, isolated from a serum sample collected from an immune pig farm in Jiangsu Province, China, in 2014. The full-genome sequence was obtained from six overlapping fragments amplified by real-time PCR (RT-PCR). The RT-PCR products were analyzed by electrophoresis through 1% agarose gels, and the target fragments were excised from the gels for purification, which was performed using a gel extraction kit (Omega, USA). Next, the purified PCR products were cloned into PMD-18-T vector (TaKaRa Dalian, China). Three recombinant clones of each fragment were sequenced by Comate Bioscience (Jilin, China). These overlapping sequences of PCR products were spliced to obtain the full-length genome of JSZL.

The complete genome sequence of JSZL is 12,297 nucleotides (nt) in length, with a 5′ UTR of 373 nt and 3′ UTR of 227 nt. A single large ORF is 11,697 nt, coding for a polyprotein of 3,898 amino acids.

In a comparison of the full-length E2 gene of JSZL with 119 reference strains, phylogenetic analysis indicated that strain JSZL belongs to the new subgenotype 2.1d (9). Furthermore, the new subgenotype has new molecular characteristics (9). Compared with the genome of subgenotype 1.1, 1.2, 2.1a, 2.1 b, 2.1c, 2.1d, 2.2, 2.3, 3.2, and 3.4, the nucleotide sequence of JSZL strain shares 84.6 to 85.4%, 85.2%, 93.1 to 94.4%, 94.6 to 96.5%, 92.2 to 92.4%, 96.6 to 97.4%, 87.6 to 91.8%, 88.8 to 89.9%, 84.0%, and 83.2 to 83.5%, respectively. Since late 2014, a large number of wild new CSFVs have been detected or isolated in many immune pig farms in China. Furthermore, most of the new CSFV isolates belong to the new subgenotype 2.1d. Therefore, these new strains and the representative strain JSZL should be worthy of high attention.

### Nucleotide sequence accession number. 

The complete genome sequence of CSFV strain JSZL has been deposited in GenBank under the accession no. KT119352.
